# Heritable variation in swimming performance in Nile tilapia (*Oreochromis niloticus*) and negative genetic correlations with growth and harvest weight

**DOI:** 10.1038/s41598-021-90418-w

**Published:** 2021-05-26

**Authors:** Samuel Bekele Mengistu, Arjan P. Palstra, Han A. Mulder, John A. H. Benzie, Trong Quoc Trinh, Chantal Roozeboom, Hans Komen

**Affiliations:** 1 Animal Breeding and Genomics, Wageningen University & Research, P.O. Box 338, 6700 AH Wageningen, The Netherlands; 2 WorldFish, Jalan Batu Maung, Batu Maung, 11960 Bayan Lepas, Penang Malaysia; 3 School of Animal and Range Sciences, College of Agriculture, Hawassa University, P. O. Box 5, Hawassa, Ethiopia; 4 School of Biological Earth and Environmental Sciences, University College Cork, Cork, Ireland

**Keywords:** Genetics, Physiology

## Abstract

Nile tilapia is predominantly produced in smallholder ponds without aeration. We hypothesize that Nile tilapia with high oxygen uptake efficiency (O_2_UE) may perform better under these conditions than Nile tilapia with low O_2_UE. Critical swimming speed (*U*_crit_, in cm s^−1^) is a potential indicator for O_2_UE. Our objectives were to estimate variance components for *U*_crit_ and fish size at swim testing early in life, and genetic correlations (*r*_*g*_) between *U*_crit_ with harvest weight (HW) and daily growth coefficient (DGC) later after grow-out in a non-aerated pond. Substantial heritability was found for absolute *U*_crit_ (0.48). The estimated *r*_*g*_ between absolute *U*_crit_ and fish size at testing were all strong and positive (range 0.72–0.83). The estimated *r*_*g*_ between absolute *U*_crit_ and HW, and absolute *U*_crit_ and DGC were − 0.21 and − 0.63 respectively, indicating that fish with higher absolute *U*_crit_ had lower growth in the non-aerated pond as compared to fish with lower absolute *U*_crit_. These results suggest a juvenile trade-off between swimming and growth performance where fish with high *U*_crit_ early in life show slower growth later under conditions of limited oxygen availability. We conclude that *U*_crit_ in Nile tilapia is heritable and can be used to predict growth performance.

## Introduction

Nile tilapia (*Oreochromis niloticus*) is predominantly produced in smallholder tilapia ponds without aeration. In non-aerated ponds, dissolved oxygen (DO) drops below critical level (3 mg l^−1^^1^) during the night. Low DO in smallholder farms negatively affects Nile tilapia growth^2^. It may be expected, therefore, that Nile tilapia with high oxygen uptake efficiency may grow better under these conditions than Nile tilapia with low oxygen uptake efficiency. As critical swimming speed (*U*_crit_) may reflect the oxygen uptake efficiency, the hypothesis is that fish with high *U*_crit_ will grow better under conditions where oxygen is limiting.

A high throughput method to assess the individual variation in oxygen uptake efficiency is by subjecting fish to exhaustive exercise in a critical swimming challenge test. In this test, swimming speeds are incrementally increased at prescribed intervals until fish stop swimming and fatigue^3,4^. Individual fish fatigue when swimming at a specific speed interval for a certain period, from which the *U*_crit_^3^ can be determined. Recently we have developed and applied such tests for gilthead seabream (*Sparus aurata*) and Atlantic salmon (*Salmo salar*)^5^. Oxygen uptake is maximal at *U*_crit_, although the anaerobic component by fast skeletal muscle increases when nearing *U*_crit_^6^. Near *U*_crit_, the metabolic demand for oxygen is becoming greater than can be provided by ventilatory and circulatory systems^7^. Fish that are able to consume more oxygen can swim faster, or reverse for the connection that we are interested in: faster swimming fish have higher oxygen uptake efficiency. Particularly for tilapia, the link between *U*_crit_ and maximal oxygen consumption may be strong because tilapia has a high *U*_crit_ (4.94 ± 0.45 BL s^−1^ for ~ 15 cm fish) and a very high maximum metabolic rate^8^. Hence, *U*_crit_ could be a good indicator of oxygen uptake efficiency of individual tilapia.

The heredity of athletic performance has received considerable research attention in dog^9^, horse^10,11^ and human^12^. Genetic parameter estimates for swimming performance in fish are scarce, but suggest that swimming performance has a heritable component. Broad sense heritabilities (i.e. not corrected for dominance and epistatic interaction effects)^13^ of swimming performance were estimated by Garenc et al.^14^ in stickleback (*Gasterosteus aculeatus*), by Hurley and Schom^15^ in Atlantic salmon and by Nicoletto^16^ in guppy (*Poecilia reticulata*). More recently, Vandeputte et al.^17^ estimated the additive genetic variance component for relative *U*_crit_ (*U*_crit_ divided by standard length) in European sea bass (*Dicentrarchus labrax*) and found a heritability of 0.55, with a negative genetic correlation with body weight.

We therefore aimed first to estimate variance components for swimming performance in Nile tilapia expressed as *U*_crit_ and to estimate the genetic correlation between *U*_crit_ and fish size at swim testing early in life. Next, tested fish were stocked in a non-aerated pond and grown to harvest weight, to determine the genetic correlations between *U*_crit_ early in life and harvest weight (HW) and daily growth coefficient (DGC) later in life.

## Materials and methods

### Ethics statement

This study utilised phenotypic data collected as part of the GIFT selective breeding program managed by WorldFish at the Aquaculture Extension Centre of the Malaysian Department of Fisheries at Jitra, Kedah State, Malaysia (6° 15′ 32° N; 100° 25′ 47° E). This study was approved by the internal WorldFish ethics committee. All fish in the GIFT breeding population are managed in accordance with the Guiding Principles of the Animal Care, Welfare and Ethics Policy of WorldFish.

### Experimental fish

Nile tilapia of the Genetically Improved Farmed Tilapia (GIFT) strain from generation 18 was used in this experiment. The 60 full sib and half sib families were produced using 31 males and 58 females, of which two females were used twice with different males. The planned mating ratios were one male to at least two females. However, the successful matings were: 12 males each mated with one female (resulting in 12 full sib families), 12 males each mated with two females (resulting in 12 half sib groups equivalent to 24 full sib families), 4 males each mated with 3 females (four half sib groups equivalent to 12 full sib families) and 3 males each mated with 4 females (three half sib groups equivalent to 12 full sib families). Each full sib family was reared separately in a hapa (fine mesh net enclosure) set up in an earthen pond.

**Figure 1:**
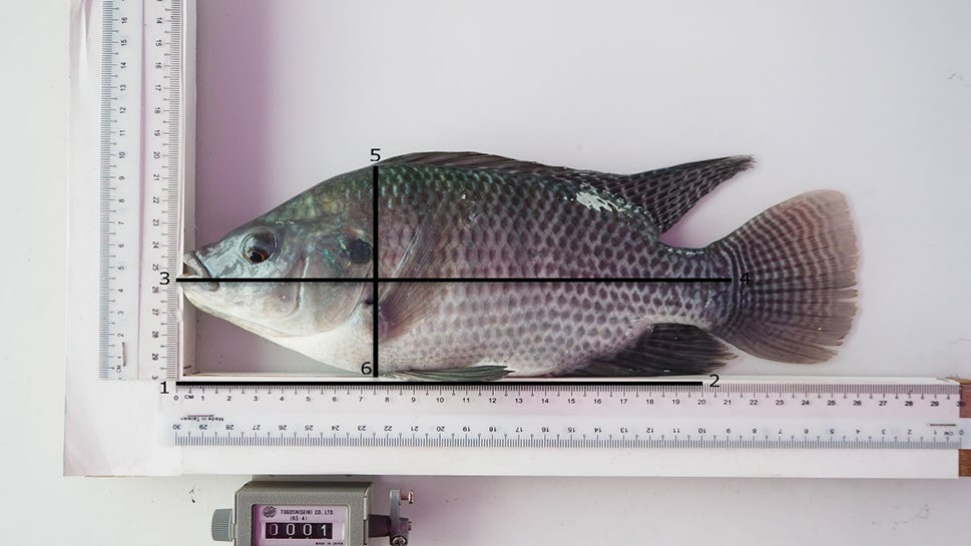
Nile tilapia with landmarks 1:6. Landmarks 1 and 2 marks a reference scale of 20 cm length, landmarks 3 and 4 represent the snout and base of the caudal fin, respectively, landmarks 5 and 6 were used to measure height (maximum dorso-ventral length) of the experimental fish.

The image analysis was done as described previously by Mengistu et al.^18^. In total 1500 photographs were loaded into tpsUtil software^19^ and digitized for six landmarks using tpsDig 2.30^20^. Landmarks 1 and 2 were on the 0 and 20 cm marks on the ruler which was photographed together with the fish for scaling. The landmarks 3 and 4 were used to measure standard length, the distance between the tip of the snout to the base of caudal fin. The landmarks 5 and 6 were the dorsal and ventral landmarks where the distance is maximum. These landmarks were used to calculate height, the maximum dorso-ventral distance (Fig. 1). To obtain the distance between the Cartesian coordinates, these landmarks were analysed in R software using Geomorph package version 3.0.7^21^ and the true distance in cm was computed based on the reference scale.

### Swim test experiment

The swim test was done in 30 working days, one swim test per day. Thirty to 35 relatively bigger fingerlings from 60 full sib families were selected, PIT tagged and housed in a tank. Three weeks after PIT tagging, 25 fish in a range from 5 to 10 cm standard length at swim testing (SLtest, in cm) from each of the 60 full sib families were measured using a ruler with a centimetre scale, weighed (Wtest, in g) and photographs were made, one day before the swim test. The SLtest and height at swim testing (Htest, in cm) of the fish used in our analysis were obtained from the photographs of each fish using image analysis. The number of fish tested per family was 25 and the number of fish per test was 50 fish. Therefore, we tested either 10 fish from 5 families or 5 fish from 10 families which resulted in all 25 fish from each family being tested in three consecutive days.

To determine the *U*_crit_, a Brett type (rectangular oval shape raceway) swim flume of 230 cm length and 90 cm width with a water depth of 40 cm was used^22^. Water current was created using a Minn Kota Terrova 80 lbs propeller. The propeller has 10 speed settings, in this experiment speed levels from 2 to 10 were used. Supplementary Table 1 provides the flow speeds measured at each of the settings. As the assessment of *U*_crit_ requires all fish to fatigue, this experimental set-up could be applied for early life testing at small size and not for older and larger fish.

Feeding was stopped 24 h before the beginning of the swim testing. The fish were acclimatised for one hour in the swimming flume without flow. After acclimation, the propeller was turned on to induce swimming at the second setting. The time at each setting was fixed at 30 min and flow increments continued until all fish fatigued. At each setting, the average water flow velocity was recorded using a FP111 Global Water Flow Probe (FP111, Global Water, USA). The swim test could take maximally 4.5 h, with 9 propeller speed levels. A fish fatigued when it touched the back fence and could not be stimulated to continue swimming. Each fatigued fish was scooped out immediately and PIT tag number and time at fatigue were recorded.

The mean DO in the tank just before resuming the swim test was 5.6 ± 0.4 mg l^−1^ (71.2% saturation), ranging from 4.9 to 6.4 mg l^−1^, and during the swim test it was 7.6 ± 0.4 mg l^−1^ (97.6% saturation), ranging from 6.4 to 8.8 mg l^−1^. The mean water temperature in the tank just before resuming the swim test was 27.7 ± 0.6 °C, ranging from 26.5 to 28.5 °C, and during the swim test it was 28.3 ± 0.6 °C, ranging from 26.5 to 29.9 °C.

### Calculation of critical swimming performance and surface area

Absolute and relative critical swimming speed (*U*_crit_) was used as a measure of swimming performance and calculated according to Brett^3^:

1 $${\text{Absolute}}\;U_{{{\text{crit}}}} = {\text{U}}_{{ - 1}} + \left( {\frac{{\text{t}}}{{\Delta {\text{t}}}}} \right)\Delta {\text{U}}$$

2 $${\text{Relative}}\;U_{{{\text{crit}}}} = \left( {{\text{U}}_{{ - 1}} + \left( {\frac{{\text{t}}}{{\Delta {\text{t}}}}} \right)\Delta {\text{U}}} \right)/{\text{SLtest}}$$

where ${\text{U}}_{ - 1}$ is the highest velocity maintained for the prescribed period in ${\text{cm}}\;{\text{s}}^{{ - 1}}$, $\Delta {\text{U}}$ is velocity increment in ${\text{cm}}\;{\text{s}}^{{ - 1}}$, $t$ is time to fatigue at final velocity level in minutes, $\Delta {\text{t}}$ is the time each velocity level is maintained at (= 30 min) and ${\text{SLtest}}$ is standard length of fish at swim testing in cm. Figures were produced using Minitab software^23^.

Surface area at swim testing (SAtest) of Nile tilapia is similar to the area of an ellipse and was calculated as:

3 $$SA = \frac{1}{4}\pi *SLtest*Htest$$

### Grow-out in the non-aerated pond

Swim tested fish were stocked in a non-aerated pond for grow-out. The pond size was 500 ${\text{m}}^{2}$ and the stocking density was 3 fish per ${\text{m}}^{2}$. During the grow-out period, DO was above 5 mg ${\text{l}}^{ - 1}$ except from 9:00 p.m. to 9:00 a.m. when DO would drop below 3 mg ${\text{l}}^{ - 1}$. Fish were weighed and photographed before stocking into the non-aerated pond. The mean weight at cultivation start (Wstart) was 10.8 g and the coefficient of variation (CV) was 23.7. The fish were fed commercial feed at a rate of 3 to 5% of their body weight depending on their sizes, with the percentage of feed decreasing with size. The fish were harvested after 145 or 146 days of grow-out. Each fish was weighed at harvest. At harvest the sex of a random half of the fish (763 fish) were determined.

Daily growth coefficient (DGC)^24,25^ was computed as:

4 $$DGC = \left[ {\frac{{\sqrt[3]{HW} - \sqrt[3]{Wstart}}}{time\, in\, days}} \right] \times 100$$

where HW is harvest weight and Wstart is stocking weight.

### Statistical analysis

Phenotypic and genetic parameters were estimated using ASReml version 4.1^26^. The following animal model was used:

5 $${\varvec{y}} = {\mathbf{Xb}} + {\mathbf{Z}}_{1} {\mathbf{a}} + {\mathbf{Z}}_{2} {\mathbf{c}} + {\mathbf{e}}$$

where ${\varvec{y}}$ is a vector of either absolute *U*_crit_, or relative *U*_crit_ in the univariate model, ${\mathbf{b}}$ is the vector of fixed effects, that is test day and sex fitted as class variable for relative *U*_crit_ while for absolute *U*_crit_ three different models were fitted with: (1) test day and sex fitted as class variables, (2) test day and sex as class variables and Wtest as a covariate and (3) test day and sex as class variables and SLtest as a covariate, sex was not significant in all the three models; therefore, sex was removed from the models; ${\mathbf{a}}$ is a vector of additive genetic effects, ${\mathbf{c}}$ is a vector of environmental effects common to full sibs (‘hapa effect’), and ${\mathbf{e}}$ a vector of residual effects. The **X**, **Z**_**1**_ and **Z**_**2**_ are design matrices assigning phenotypic values to the levels of fixed effect, additive genetic and common environmental effects, respectively. The effect of sex was also not significant when subset of the data with only 763 sexed fish was analysed.

Bivariate models were used to estimate the phenotypic and genetic correlations between absolute *U*_crit_, and traits such as Wtest, SLtest, Htest, SAtest, HW and DGC. In the bivariate models test day and sex were fitted as a class variable for absolute *U*_crit_, age at harvest was fitted as a covariate for HW and sex was fitted as a class variable for DGC. Common environmental effect was fitted as a random variable to all the traits except for DGC in the bivariate model absolute *U*_crit_ and DGC. The bivariate model with absolute *U*_crit_ and DGC did not converge when a common environmental effect was fitted as a random effect on both traits. The additive genetic effects were normally distributed as $${\text{N}} = \left( {\left[ {\begin{array}{*{20}c} 0 \\ 0 \\ \end{array} } \right],{ }{\mathbf{A}} \otimes \left[ {\begin{array}{*{20}c} {\sigma_{a,1}^{2} } & {r_{a,12} \sigma_{a,1} \sigma_{a,2} } \\ {r_{a,21} \sigma_{a,2} \sigma_{a,1} } & {\sigma_{a,2}^{2} } \\ \end{array} } \right]} \right)$$, where ${\mathbf{A}}$ is the numerator genetic relationships matrix and ${\upsigma }_{{{\text{a}},1}}^{2}$ (${\upsigma }_{{{\text{a}},2}}^{2}$) being the additive genetic variance of trait 1(2) and $r_{{a,12\left( {21} \right)}}$ being the genetic correlation between trait 1 and 2. The pedigree depth was 18 generations, i.e. from the current generation G18 all the way back to the first generation of GIFT in WorldFish, Malaysia. The common environmental effects were normally distributed as $${\text{N}} = \left( {\left[ {\begin{array}{*{20}c} 0 \\ 0 \\ \end{array} } \right],\user2{ }{\mathbf{I}} \otimes \left[ {\begin{array}{*{20}c} {\sigma_{c,1}^{2} } & {r_{c12} \sigma_{c,1} \sigma_{c,2} } \\ {r_{c21} \sigma_{c,2} \sigma_{c,1} } & {\sigma_{c,2}^{2} } \\ \end{array} } \right]} \right)$$, where **I** being an identity matrix and ${\upsigma }_{{{\text{c}},1}}^{2}$ (${\upsigma }_{{{\text{c}},2}}^{2}$) being the common environmental variance of trait 1(2) and $r_{{c,12\left( {21} \right)}}$ being the common environmental correlation between trait 1 and 2. The residual effects were normally distributed as $${\text{N}} = \left( {\left[ {\begin{array}{*{20}c} 0 \\ 0 \\ \end{array} } \right],\user2{ }{\mathbf{I}} \otimes { }\left[ {\begin{array}{*{20}c} {{\upsigma }_{{\text{e,1}}}^{{2}} } & {{\text{r}}_{{\text{e, 12}}} {\upsigma }_{{\text{e,1}}}^{{2}} {\upsigma }_{{\text{e,2}}}^{{2}} } \\ {{\text{r}}_{{\text{e,21}}} {\upsigma }_{{\text{e,2}}}^{{2}} {\upsigma }_{{\text{e, 1}}}^{{2}} } & {{\upsigma }_{{\text{e,2}}}^{{2}} } \\ \end{array} } \right]} \right)$$, where ${\upsigma }_{{{\text{e}},1}}^{2}$ (${\upsigma }_{e,2}^{2}$) being the residual variance of trait 1(2) and $r_{{e,12\left( {21} \right)}}$ being the residual correlation between trait 1 and 2.

Heritability $\left( {{\text{h}}^{2} } \right)$ and the ratio of common environmental variance $({\text{c}}^{2}$) to phenotypic variance ($\sigma_{p}^{2} )$ of each trait was computed as ${\text{h}}^{2} = {\upsigma }_{{\text{a}}}^{2} /{\upsigma }_{{\text{p}}}^{2}$ and ${\text{c}}^{2} = {\upsigma }_{{\text{c}}}^{2} /{\upsigma }_{{\text{p}}}^{2}$, respectively. The significance of the random effects were tested using loglikelihood ratio test with one degree of freedom^27^. To test whether the genetic correlation is larger than zero, a model without constraining the covariance was tested against a model where the covariance was constrained to zero. The full model, i.e. a model with both common environmental effects and additive genetic effects as random effects, was tested against a reduced model, i.e. a model with either only common environmental effect or additive genetic effects as a random effect. The common environmental variances were not significantly different from zero (*P* > 0.05) except for relative *U*_crit_ (*P* = 0.006). The most likely reason that the common environmental effect was not significant in most cases was because of the almost complete confounding of sire genetic, dam genetic and common environmental effects in the experiment. This reflected the fact that 24 of the males were mated to one or two females resulting in 12 families with no half sib families and 12 families with only one half sib family (40% of the total families), making genetic and common environmental effects difficult to disentangle. Although, common environmental effects were not significant for most traits, common environmental effects explained a substantial part of the phenotypic variance and were kept in the model, to prevent overestimation of the additive genetic variance. The loglikelihood for the bivariate model with absolute *U*_crit_ and DGC did not converge when common environmental effect was fitted as a random effect on both traits. Therefore, the common environmental effect was fitted as a random effect only on absolute *U*_crit_ in the bivariate model with absolute *U*_crit_ and DGC.

## Results

### Biometric data

In total 1500 fish were swim tested and stocked in the non-aerated pond. Out of the swim tested 1500 fish, the swimming performance data of seven fish were missing and resulted in 1493 *U*_crit_ records. The descriptive statistics for age at swim testing (Agetest), Wtest, SLtest, Htest, SAtest, weight at cultivation start, and HW and DGC later in life are presented in Table 1. Out of the stocked 1500 fish, ultimately 1199 were harvested which is equivalent to 79.9% survival.

**Table 1:** Number of fish (N), mean, standard deviation (SD), coefficient of variation (CV) and minimum and maximum values for critical swimming speed (*U*_crit_), absolute and relative, age at swim testing (Agetest), body weight at swim testing (Wtest), standard length at swim testing (SLtest), and body height at swim testing (Htest), surface area at swim testing (SAtest), weight at cultivation start(Wstart), harvest weight (HW) and daily growth coefficient (DGC).

	N	Mean	SD	CV	Min	Max
Absolute *U*_crit_ (cm s^−1^)	1493	69.1	5.5	7.9	50.6	83.8
Relative *U*_crit_ (SL s^−1^)	1493	9.7	0.9	9.8	6.9	13.3
Agetest (days)	1500	86.8	12.1	13.9	65	139
Wtest (g)	1500	10.8	2.6	23.7	4.8	20.1
SLtest (cm)	1500	7.2	0.6	8.0	5.3	8.9
Htest (cm)	1500	2.7	0.2	9.0	2.1	3.5
SAtest (cm^2^)	1500	15.3	2.5	16.4	9.2	24.0
Wstart (g)	1199	27.4	15.1	47.8	7.3	94.2
HW (g)	1199	417.7	88.1	21.1	153.4	778.9
DGC	1199	3.1	0.3	10.8	1.7	3.8

### Swimming performance

**Figure 2:**
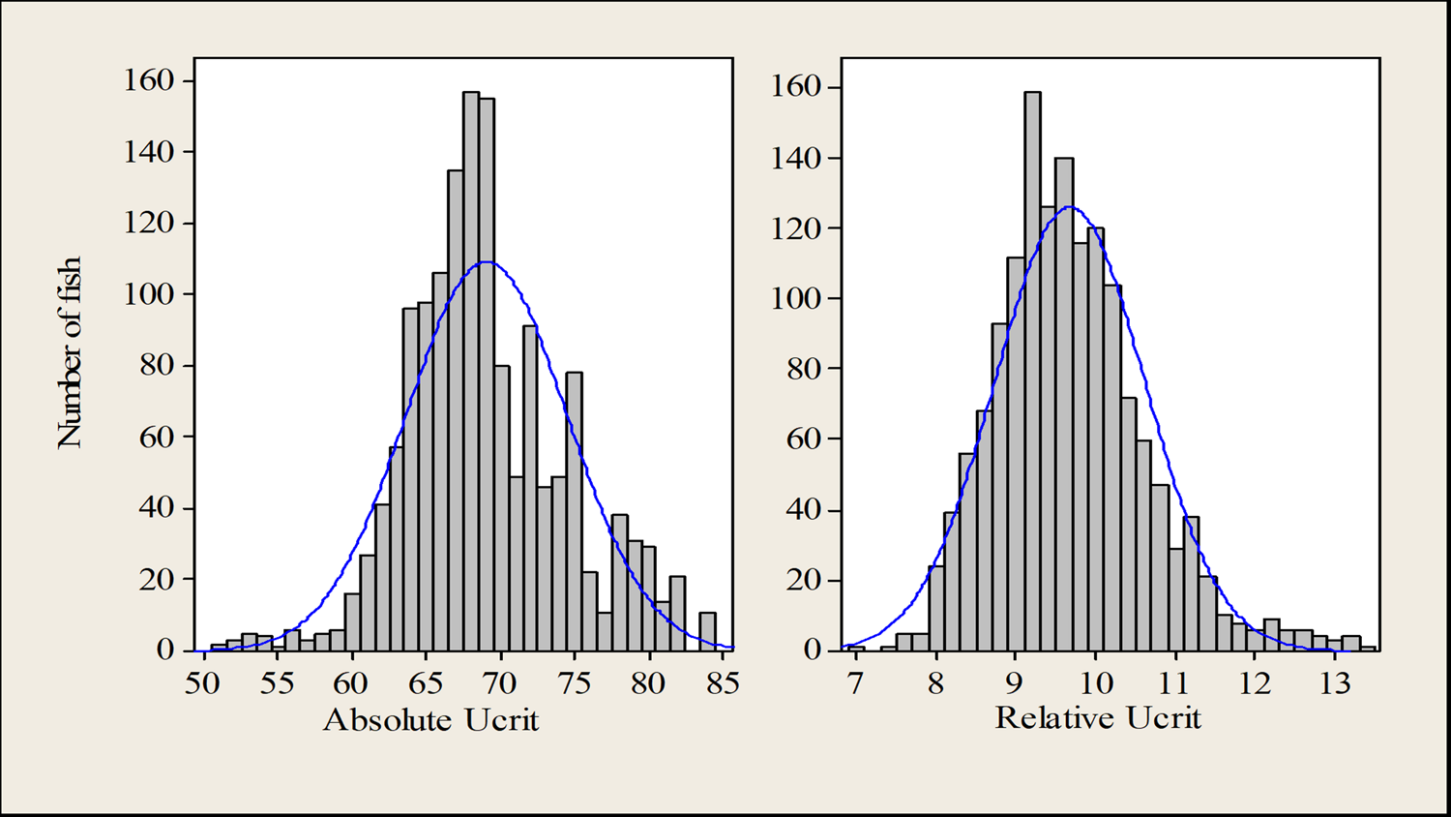
Distribution of absolute *U*_crit_ (cm s^−1^) and relative *U*_crit_ (SL s^−1^).

**Figure 3:**
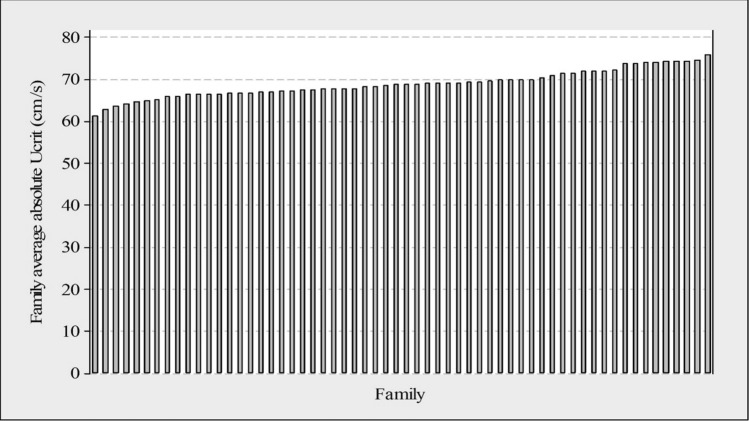
Histogram of family average absolute *U*_crit_ (cm s^−1^) for each of the 60 families.

The mean absolute *U*_crit_ and relative *U*_crit_ were 69.1 ± 5.5 cm s^−1^ and 9.7 ± 0.9 SL s^−1^, respectively (Table 1). Absolute *U*_crit_ and relative *U*_crit_ values showed normal distributions (Fig. 2). There was substantial variation in swimming performance between family means (Fig. 3), indicating existence of genetic variation.

### Genetic parameters

Variances, heritability and the ratio of common environmental variance to the phenotypic variance (c^2^) effect for absolute and relative *U*_crit_ are presented in Table 2. The heritability for absolute *U*_crit_ was 0.48 ± 0.17 when Wtest or SLtest was not fitted in the model as a covariate. The heritability for absolute *U*_crit_ was 0.41 ± 0.16 when SLtest was fitted as a covariate and 0.44 ± 0.16 when Wtest was fitted as a covariate. The heritability for relative *U*_crit_ (0.15 ± 0.13) was low. The common environmental effect explained a small proportion of the phenotypic variance (0.02 to 0.04) for absolute *U*_crit_, while the contribution was substantial for the phenotypic variance of relative *U*_crit_ (0.13). The analyses with absolute *U*_crit_ with Wtest or SLtest in the model as a covariate showed that *U*_crit_ contained considerable heritable variation even when corrected for body size.

**Table 2:** Additive genetic variance (${\sigma }_{a}^{2}$), common environmental variance (${\sigma }_{c}^{2}$), phenotypic variance (${\sigma }_{p}^{2}$), heritability and common environmental effect (${c}^{2}$) of absolute and relative critical swimming speed (*U*_crit_).

	${\sigma }_{a}^{2}$	${\sigma }_{c}^{2}$	${\sigma }_{p}^{2}$	Heritability	c^2^
Absolute *U*_crit_*	8.90	0.43	18.45	0.48 ± 0.17	0.02 ± 0.05
Absolute *U*_crit_**	6.71	0.59	16.20	0.41 ± 0.16	0.04 ± 0.05
Absolute *U*_crit_***	6.79	0.55	16.09	0.44 ± 0.16	0.03 ± 0.05
Relative *U*_crit_	0.08	0.07	0.55	0.15 ± 0.13	0.13 ± 0.06

*Absolute *U*_crit_ without body weight or standard length at swim testing in the model.

**Absolute *U*_crit_ when standard length at swim testing was included in the model as covariate.

***Absolute *U*_crit_ when body weight at swim testing was included in the model as covariate.

The additive genetic variance contributed a significant proportion to the phenotypic variance of absolute *U*_crit_ (*P* = 0.000) and absolute *U*_crit_ when either Wtest (*P* < 0.001) or SLtest (*P* = 0.001) was fitted in the model as covariate, while the contribution was not significant for relative *U*_crit_ (*P* = 0.175). The contribution of common environmental effect to the phenotypic variance of absolute *U*_crit_ (*P* = 0.584) and for absolute *U*_crit_ when either Wtest (*P* = 0.384) or SLtest were fitted as covariates (*P* = 1.000) were not significant, while the contribution to the phenotypic variance of relative *U*_crit_ (*P* = 0.007) was significant.

The estimated genetic correlations (*r*_*g*_) and phenotypic correlations (*r*_*p*_) between absolute *U*_crit_ and Wtest, SLtest, Htest, SAtest, HW and DGC are presented in Table 3. The genetic correlations were significant (*P* < 0.05) except for the genetic correlation between *U*_crit_ early in life and HW later in life (*P* = 0.507) based on likelihood ratio test^27^. The estimated *r*_*g*_ and *r*_*p*_ correlations between absolute *U*_crit_ and Wtest were 0.78 and 0.44, respectively. The less than one *r*_*g*_ between absolute *U*_crit_ and Wtest indicates the presence of genetic variance in absolute *U*_crit_ that is not explained by Wtest. Genetic and phenotypic correlations with the other size measurements SLtest, Htest and SAtest were very similar. Fish with higher absolute *U*_crit_ had lower HW and DGC after grow-out in a non-aerated pond. The estimated *r*_*g*_ and *r*_*p*_ between absolute *U*_crit_ and HW were − 0.21 and − 0.04, respectively and the estimated *r*_*g*_ and *r*_*p*_ between absolute *U*_crit_ and DGC were − 0.63 and − 0.24, respectively. The negative genetic correlations between *U*_crit_ and HW and between absolute *U*_crit_ and DGC indicate that fish with higher absolute *U*_crit_ perform less in terms of HW and DGC compared to fish with lower absolute *U*_crit_.

**Table 3:** Genetic and phenotypic correlations between absolute critical swimming speed (*U*_crit_) and body weight at swim testing (Wtest), standard length at swim testing (SLtest) height at swim testing (Htest), surface area at swim testing (SAtest), harvest weight (HW) and daily growth coefficient (DGC).

	*r*_*g*_	*r*_*p*_
Wtest	0.78 ± 0.18	0.44 ± 0.05
SLtest	0.83 ± 0.19	0.43 ± 0.05
Htest	0.72 ± 0.22	0.37 ± 0.05
SAtest	0.83 ± 0.18	0.42 ± 0.05
HW	− 0.21 ± 0.29	− 0.04 ± 0.06
DGC	− 0.63 ± 0.15	− 0.24 ± 0.07

*U*_crit_ was estimated in a bivariate model without Wtest or SLtest as covariate.

## Discussion

Our objectives were to estimate variance components for swimming performance in Nile tilapia, assessed as critical swimming speed (*U*_crit_) early in life, and to estimate the genetic correlation between *U*_crit_ and body size early in life, and harvest weight (HW) and Daily Growth Coefficient (DGC) later in life after a grow-out period in a non-aerated pond. For the first time, we show with a large-scale experiment that swimming performance is heritable in Nile tilapia, and that the genetic correlation with harvest weight is strongly negative, even when corrected for body size at testing. Heritabilities, the genetic correlations, methodology and the practical application of a swimming performance test in breeding programs are discussed.

This study shows the existence of heritable variation in critical swimming performance with a moderate heritability of 0.41–0.48. Our heritability estimate for *U*_crit_ early in life is in the same range as reported previously for other species and for similar traits (for summary see Table 4). Of the four studies that estimated genetic parameters for swimming performance in fish, only the study that assessed the burst swimming performance trait is not comparable with *U*_crit_ in our study^14^. Our heritability estimate for relative *U*_crit_ (0.15) was not significantly different from zero, which is different from the heritability of 0.55 for relative maximum swimming speed in European sea bass *Dicentrarchus labrax*^17^. The difference in heritability of relative *U*_crit_ might be due to a species specific difference, particularly reflecting the high or long body shape of tilapia and seabass, respectively.

**Table 4:** Summary of the studies that estimated heritability for different swimming performance traits, genetic phenotypic correlation between swimming performance and body weight, and between swimming performance and body length.

Trait	Species	Comments	Heritability	Genetic (*r*_*g*_) and phenotypic (*r*_*p*_) correlations	References
Critical Swimming speed	Guppy (*Poecilia reticulata*)	Measured by increasing the water velocity every 3 min until the fish fatigued 16 full sib families were used (96 fish in total)	0.24 ± 0.19	Not given	^16^
Swimming stamina (similar trait with critical swimming speed)	Atlantic salmon (*Salmo salar*)	Measured as the total time the fish swam until fatigue by increasing water velocity incrementally every 4 min 11 full sib families were used (129 fish in total)	0.24 ± 0.16	*r*_*g*_ = 0.23 and *r P* = 0.85 (between stamina and body weight) *r*_*g*_ = − 0.14 and *r P* = 0.18 (between stamina and body length)	^15^
Absolute burst swimming (cm/s) (not comparable with critical swimming speed)	Threespine stickleback (*Gasterosteus aculeatus*)	Measured as distance swam in 160 ms using video recording, 2 months old 193 fish from 25 full sib families were used	0.41*	Not given	^14^
Relative burst swimming (body length/s) (not comparable with critical swimming speed)	Threespine stickleback	Measured as distance swam in 160 ms using video recording 2 months old 193 fish from 25 full sib families were used	0.37	Not given	^14^
Absolute burst swimming (cm/s) (not comparable with critical swimming speed)	Threespine stickleback	Measured as distance swam in 160 ms using video recording 3.6 months old 181 fish from 25 full sib families were used	0.02	Not given	^14^
Relative burst swimming (body length/s) (not comparable with critical swimming speed)	Threespine stickleback	Measured as distance swam in 160 ms using video recording 3.6 months old 181 fish from 25 full sib families were used	0.00	Not given	^17^
Relative maximum sustained speed (similar trait with relative critical swimming speed)	European sea bass (*Dicentrarchus labrax*)	Measured as the last fully accomplished water velocity 547 fish from 366 full sib families, paternal and maternal half sib families were used	0.55	*r*_*g*_ = − 0.64 and *r P* = − 0.56 between relative maximum sustained speed and body weight	^17^
Absolute critical swimming speed	Nile tilapia (*Oreochromis niloticus*)	Explained in Sect. 2.3 of this paper 1493 fish, full sib and half sib families Absolute *U*_crit_ without including body weight/standard length in the model as covariate	0.48	*r*_*g*_ = 0.87 and *r P* = 0.44 between absolute *U*_*crit*_ and body weight	This study
Absolute critical swimming speed	Nile tilapia	1493 fish, full sib and half sib families Absolute *U*_crit_ when body weight was included in the model as covariate	0.42		This study
Absolute critical swimming speed	Nile tilapia	1493 fish, full sib and half sib families Absolute *U*_crit_ when standard length was included in the model as covariate	0.41		This study
Relative critical swimming speed	Nile Tilapia	1493 fish, full sib and half sib families	0.15		This study

Species specific differences also exist in the relation between *U*_crit_ and body size. Absolute *U*_crit_ was genetically strongly correlated with body weight at swim testing (0.78). This is higher than the estimated genetic correlation between swimming stamina and body weight in Atlantic salmon (0.23)^15^. The genetic correlation between absolute *U*_crit_ and standard length (0.83) was also different from the estimated *r*_*g*_ between swimming stamina and fork length in Atlantic salmon (− 0.14)^15^.

To the best of our knowledge there are no studies on *r*_*g*_ between absolute *U*_crit_ and traits such as Htest, SAtest, HW and DGC with which to compare our results. In our study, the *r*_*g*_ estimates between absolute *U*_crit_ and Htest, between absolute *U*_crit_ and SAtest were 0.72 and 0.83, respectively. These strong genetic correlations between absolute *U*_crit_ and SLtest and Wtest early in life show that larger fish swim faster in absolute terms.

The estimated *r*_*g*_ values between absolute *U*_crit_ and HW and absolute *U*_crit_ and DGC were − 0.21 and − 0.63, respectively, meaning that fish with high *U*_crit_ at testing had lower growth rate (DGC) and harvest weight (HW) later in life. These negative genetic correlations do not support our hypothesis that Nile tilapia with higher *U*_crit_, reflecting higher oxygen uptake efficiency, are those that perform better in terms of weight increase in non-aerated ponds where hypoxia is frequent. Instead, the negative *r*_*g*_ shows that fish with higher *U*_crit_ early in life show less body weight increase later in life. These data do not provide insight on fish body shape and composition at slaughter size. For example, it may be that fish with higher *U*_crit_ are the leaner fish later as compared to fish with lower *U*_crit_. Fish with lower *U*_crit_ may be heavier but not necessarily have more fillet mass. Results of a *U*_crit_ test in Gilthead seabream (*Sparus aurata*), also a high bodied fish, showed that the (residual) *U*_crit_ was negatively correlated with fillet mass suggesting that fast swimmers build lower fillet mass later in life^5^. A plausible explanation for our results may be the existence of a juvenile trade-off between swimming and growth performance where fish with high *U*_crit_ early in life show slower growth later. Young juveniles may choose to either swim fast or grow fast, which may represent, for instance, two anti-predator strategies: to be able to escape predators or to become too large to be eaten rapidly. Studies have shown that a trade-off between growth rate and locomotor performance can exist^28^, for instance during accelerated growth^29^ which can negatively influence muscle cellularity and development^30,31^. Indeed, fast-growing growth hormone (GH) transgenic carp^32^ had lower critical swimming performance than non-transgenic controls. Fast-growing GH transgenic salmon had similar critical swimming speeds than non-transgenic controls but were also able to consume considerable more oxygen^33^ and may thus have compensated for lower critical swimming performance.

In our study, 1,493 fish were used to estimate genetic parameters. The mating ratio used to produce the experimental fish was 1 male to 1 – 4 females, which gave full sib and half sib families. The previous studies that estimated genetic parameters used a much lower number of fish (range 96–129) as compared to our study and estimated broad sense heritability using full sib families (Table 4)^15,16^. The much larger sample size gave a much higher precision of estimates of narrow-sense heritability. Furthermore, broad-sense heritability estimates are biased estimates of narrow-sense heritabilities, because broad-sense heritabilities contain non-additive genetic variation due to dominance and epistasis that is not heritable from parent to offspring and may contain common environmental effects, because in such full-sib designs estimation of common environmental effects is not feasible^34^. Narrow-sense heritability, however, is the ratio of additive genetic variance to phenotypic variance^13^ and therefore a better indication of the proportion of genetic variation that is transmitted to the next generation. In our study, we used half sib families that enabled us to estimate a narrow sense heritability. Similarly, Vandeputte et al.^17^ estimated narrow sense heritability using half sib families based on 547 fish. The main difference in the swimming performance trait between our study and Vandeputte et al.^17^ was that these authors did not include the last water velocity level that the fish did not fully complete. Besides the species difference mentioned earlier, also the number of fish and the way the swimming performance was calculated could provide additional explanation for the difference in the parameter estimates between our study and Vandeputte et al.^17^.

Critical swimming speed can be calculated in four different ways: as absolute *U*_crit_, with or without Wtest or SLtest as covariate in the model, as relative *U*_crit_, or as residual *U*_*crit*_ which is the difference in *U*_crit_ of an individual fish with the predicted value on basis of its length^5^. Analysing absolute *U*_crit_ without a covariate for either Wtest or SLtest, has the highest additive genetic variance, but part of that genetic variance is due to genes affecting body size. The use of fish with similar body weight at similar SLtest is practically difficult as the variation is considerable; in our experiment the Wtest was from 4.8 to 20.1 g for fish from 5.4 to 10 cm SL. Therefore, it is important to account for Wtest or SLtest in the analysis to be able to estimate heritable variation in *U*_crit_ independent of body size.

Relative *U*_crit_ is a ratio of *U*_crit_ to SLtest for which the estimated heritability was not significantly different from zero in our study. Relative *U*_crit_ is a ratio trait and therefore the genetic variance becomes a complex function of absolute *U*_crit_ and SLtest. Ratio traits are generally not recommended in animal breeding^35^. For instance, the heritability of a ratio trait cannot be used to predict the genetic change for the ratio trait^36^. Therefore, we recommend using the absolute *U*_crit_ and to fit either Wtest or SLtest as a covariate in a model when estimating heritability. Such an analysis shows the existence of heritable variation in *U*_crit_ beyond body size.

The less than unity genetic correlation between absolute *U*_crit_ and Wtest indicates the presence of genetic variation in *U*_crit_, independently of Wtest. A genetic correlation of unity between two traits means that the two traits are controlled by the same genes while a genetic correlation of less than unity indicates that there are additional genes that are not common for the two traits and only control one of the two traits. The negative *r*_*g*_ between absolute *U*_crit_ and HW, and between *U*_crit_ and DGC, clearly indicates that selection for high harvest weight will favour faster growing animals with lower *U*_crit_. Whether this is desirable needs to be determined. One can speculate that under conditions of hypoxia, as frequently encountered in non-aerated ponds or ponds with algal blooms, smaller, more active fish will have a higher chance of survival. In optimal management conditions, however, growth rate can be further increased by including *U*_crit_ at testing in the breeding goal, next to harvest weight. Fish with higher *U*_crit_ may also be more resilient: swimming exercise improves physiological fitness; cardiovascular and respiratory performance, and increases mitochondrial densities and muscle tissue capillarization^37^. Also the immune system capacity appears to be linked to swimming performance as Castro et al.^38^ found 21 virus-responsive genes with significantly higher transcript abundance in phenotypically poor swimmers as compared to good swimmers in Atlantic salmon.

In conclusion, including absolute *U*_crit_ in a breeding goal in addition to HW and DGC could be beneficial if the aim is to select for fitter fish, especially in environments where oxygen is limiting. Absolute *U*_crit_ can be measured at an early stage on the selection candidates themselves, high throughput and non-invasively although size of the tested fish may be restricted due to difficulty in reaching sufficiently high flow speed. However, selection on *U*_crit_ with 10% selection intensity for the highest values of *U*_crit_ could lead to a 19% reduction in mean harvest weight of the offspring, compared to direct selection on harvest weight. In practice we recommend a two-stage selection scheme, where selection in the first stage is on retaining 90% of the fittest fish in terms of *U*_crit_, followed by a second stage selection on harvest weight. This study showed for the first time the existence of significant additive genetic variance for critical swimming speed in Nile tilapia. Favourable *r*_*g*_ between *U*_crit_ and traits such as Wtest, SLtest, Htest and SAtest early in life were found. The main finding demonstrated a negative *r*_*g*_ between *U*_crit_ and HW later in life, and between *U*_crit_ and DGC later in life. Including *U*_crit_ in the breeding goal may help to improve resilience of Nile tilapia.

## Supplementary Information

Supplementary Information.

## Data Availability

Data are available from the corresponding author upon reasonable request.

## References

[1] Stickney RR (2017). Aquaculture: An Introductory Text.

[2] Mengistu SB, Mulder HA, Benzie JAH, Komen H (2020). A systematic literature review of the major factors causing yield gap by affecting growth, feed conversion ratio and survival in Nile tilapia (*Oreochromis niloticus*). Rev. Aquacult..

[3] Brett JR (1964). The respiratory metabolism and swimming performance of young sockeye salmon. J. Fish. Res. Board Can..

[4] Plaut I (2000). Effects of fin size on swimming performance, swimming behaviour and routine activity of zebrafish *Danio rerio*. J. Exp. Biol..

[5] Palstra AP, Kals J, Böhm T, Bastiaansen J, Komen H (2020). Swimming performance and oxygen consumption as non-lethal indicators of production traits in Atlantic salmon and Gilthead seabream. Front. Physiol..

[6] Videler JJ (1993). Fish Swimming.

[7] Jones DR, Randall DJ, Hoar WS, Randall DJ (1978). Respiration and circulation during exercise in fish. Fish Physiology.

[8] McKenzie DJ, Martínez R, Morales A, Acosta J, Morales R, Taylor EW (2003). Effects of growth hormone transgenesis on metabolic rate, exercise performance and hypoxia tolerance in tilapia hybrids. J. Fish Biol..

[9] Kim J, Williams FJ, Dreger DL, Plassais J, Davis BW, Parker HG, Ostrander EA (2018). Genetic selection of athletic success in sport-hunting dogs. Proc. Nat. Acad. Sci. USA.

[10] Hellsten T, Viklund Å, Koenen EPC, Ricard A, Bruns E, Philipsson J (2006). Review of genetic parameters estimated at stallion and young horse performance tests and their correlations with later results in dressage and show-jumping competition. Livest. Sci..

[11] Hill EW, Ducro BJ, van Weeren R, Barneveld A, Back W, Back W, Clayton HM (2013). Genetic contributions to exercise and athletic performance. Equine Locomotion.

[12] Issurin VB (2017). Evidence-based prerequisites and precursors of athletic talent: A review. Sports Med..

[13] Falconer DS, Mackay TFC (1996). Introduction to Quantitative Genetics.

[14] Garenc C, Silversides FG, Guderley H (1998). Burst swimming and its enzymatic correlates in the threespine stickleback (*Gasterosteus aculeatus*): Full-sib heritabilities. Can. J. Zool..

[15] Hurley SM, Schom CB (1984). Genetic control of swimming stamina in Atlantic salmon (*Salmo salar*). Can. J. Genet. Cytol..

[16] Nicoletto PF (1995). Offspring quality and female choice in the guppy, *Poecilia reticulata*. Anim. Behav..

[17] Vandeputte M, Porte JD, Auperin B, Dupont-Nivet M, Vergnet A, Valotaire C (2016). Quantitative genetic variation for post-stress cortisol and swimming performance in growth-selected and control populations of European sea bass (*Dicentrarchus labrax*). Aquaculture.

[18] Mengistu SB, Mulder HA, Benzie JAH, Khaw HL, Megens TTQ (2020). Genotype by environment interaction between aerated and non-aerated ponds and the impact of aeration on genetic parameters in Nile tilapia (*Oreochromis niloticus*). Aquaculture.

[19] Rohlf, F. J. *tpsUtil, Version 1.74*. (Department of Ecology and Evolution, State University of New York at Stony Brook, 2017).

[20] Rohlf, F. J. *tpsDig2, Version 2.30*. (Department of Ecology and Evolution, State University of New York at Stony Brook, 2017).

[21] Adams, D. C., Collyer, M. L., Kaliontzopoulou, A. *Geomorph: Software For Geometric Morphometric Analyses. *R package Version 3.0.6. (2018).

[22] Palstra, A. P. *Physiological Testing of Fish in WorldFish Facilities in Malaysia. Design of Swim Flume and Standard Operating Procedure for High Throughput Testing of Juvenile Tilapia*. *Confidential Report 516* pp. 16 (Wageningen Livestock Research, 2016).

[23] Minitab Statistical Software Version 17. (2010). https://www.minitab.com

[24] Iwama GK, Tautz AF (1981). A simple growth model for salmonids in hatcheries. Can. J. Fish. Aquat. Sci..

[25] Trọng TQ, Mulder HA, van Arendonk JAM, Komen H (2013). Heritability and genotype by environment interaction estimates for harvest weight, growth rate, and shape of Nile tilapia (*Oreochromis niloticus*) grown in river cage and VAC in Vietnam. Aquaculture.

[26] *ASReml User Guide Release 4.1 Functional Specification* (2015).

[27] Lynch M, Walsh B (1997). Genetics and Analysis of Quantitative Traits.

[28] Billerbeck JM, Lankford TE, Conover DO (2001). Evolution of intrinsic growth and energy acquisition rates. I. Tradeoffs with swimming performance in *Menidia menidia*. Evolution.

[29] Lee W-S, Monaghan P, Metcalfe NB (2010). The trade-off between growth rate and locomotor performance varies with perceived time until breeding. J. Exp. Biol..

[30] Galloway TF, Kjorsvik E, Kryvi H (1999). Muscle growth and development in Atlantic cod larvae (*Gadus morhua* L.) related to different somatic growth rates. J. Exp. Biol..

[31] Johnston IA (2003). Muscle metabolism and growth in Antarctic fishes (suborder Notothenioidei): Evolution in a cold environment. Comp. Biochem. Physiol. B Biochem. Mol. Biol..

[32] Li D, Fu C, Hu W, Zhong S, Wang Y, Zhu Z (2007). Rapid growth cost in “all-fish” growth hormone gene transgenic carp: Reduced critical swimming speed. Chin. Sci. Bull..

[33] Stevens ED, Sutterlin A, Cook T (1998). Respiratory metabolism and swimming performance in growth hormone transgenic Atlantic salmon. Can. J. Fish. Aquat. Sci..

[34] Lozano-Jaramillo M, Komen H, Wientjes YCJ, Mulder HA, Bastiaansen JWM (2020). Optimizing design to estimate genetic correlations between environments with common environmental effects. J. Anim. Sci..

[35] Zetouni L, Henryon M, Kargo M, Lassen J (2017). Direct multitrait selection realizes the highest genetic response for ratio traits. J. Anim. Sci..

[36] Gunsett FC (1987). Merit of utilizing the heritability of a ratio to predict the genetic change of a ratio. J. Anim. Sci..

[37] Palstra AP, Planas JV (2011). Fish under exercise. Fish Physiol. Biochem..

[38] Castro V, Grisdale-Helland B, Jørgensen SM, Helgerud J, Claireaux G, Farrell AP (2013). Disease resistance is related to inherent swimming performance in Atlantic salmon. BMC Physiol..

