# Supplementary material for: Heritable variation in swimming performance in Nile tilapia (Oreochromis niloticus) and negative genetic correlations with growth and harvest weight
Source: Sci Rep. 2021 May 26;11:11018. doi: 10.1038/s41598-021-90418-w (PMC8154888; doi:10.1038/s41598-021-90418-w)
Supplement: Supplementary file 1 — Supplementary Information. [file 41598_2021_90418_MOESM1_ESM.pdf]

Heritable variation in swimming performance in Nile tilapia (*Oreochromis niloticus*) and negative genetic correlations with growth and harvest weight

Samuel Bekele Mengistu[a, b, c], Arjan P. Palstra[a, *], Han A. Mulder[a], John A. H. Benzie[b,d], Trong Quoc Trinh[d], Chantal Roozeboom[a], Hans Komen[a]

[a]Wageningen University & Research Animal Breeding and Genomics, P.O. Box 338, 6700 AH Wageningen, The Netherlands

[b]WorldFish, Jalan Batu Maung, Batu Maung, 11960 Bayan Lepas, Penang, Malaysia

[c]School of Animal and Range Sciences, College of Agriculture, Hawassa University, P. O. Box 5, Hawassa, Ethiopia

[d]School of Biological Earth and Environmental Sciences, University College Cork, Cork Ireland

*Corresponding author

E-mail: AP: arjan.palstra@wur.nl

Supplementary Table 1: Mean water velocity (cm s^{-1}) and standard deviation at different each propeller speed level.

Propeller speed level	Mean velocity cm s^{-1}	Standard deviation
2	18.60	1.25
3	31.32	1.61
4	41.83	1.39
5	51.97	1.70
6	59.99	2.48
7	66.60	3.65
8	72.90	4.67
9	77.62	5.82
10	80.84	7.89
